# Plasma Leukocyte Cell-Derived Chemotaxin-2 as a Risk Factor of Sarcopenia: Korean Frailty and Aging Cohort Study

**DOI:** 10.3390/nu17081342

**Published:** 2025-04-14

**Authors:** Eun Roh, Soon Young Hwang, Miji Kim, Chang Won Won, Kyung Mook Choi

**Affiliations:** 1 Department of Internal Medicine, Seoul National University Boramae Medical Center, Seoul 07061, Republic of Korea; 2 Department of Biostatistics, Korea University College of Medicine, Seoul 08308, Republic of Korea; 3 Department of Health Sciences and Technology, Kyung Hee University College of Medicine, Seoul 02447, Republic of Korea; 4 Elderly Frailty Research Center, Department of Family Medicine, Kyung Hee University College of Medicine, Seoul 02447, Republic of Korea; 5 Division of Endocrinology and Metabolism, Department of Internal Medicine, Korea University Guro Hospital, Korea University College of Medicine, Seoul 08308, Republic of Korea

**Keywords:** leukocyte cell-derived chemotaxin 2, non-alcoholic fatty liver disease, skeletal muscle, muscle strength, sarcopenia

## Abstract

**Background/Objective:** Leukocyte cell-derived chemotaxin-2 (LECT2), a hepatokine, is implicated in non-alcoholic fatty liver disease (NAFLD). Although NAFLD and sarcopenia are closely linked, the relationship between plasma LECT2 levels and sarcopenia remains unclear. **Methods:** We analyzed plasma LECT2 levels in 400 older adults aged 70–84 years old living in the community enrolled in the Korean Frailty and Aging Cohort Study. The appendicular skeletal muscle mass (ASM) and handgrip strength (HGS), both adjusted for the BMI, were used to evaluate the muscle mass and strength. A low muscle mass (LMM) was defined using the sex-specific lowest quintile of ASM/BMI as the cutoff value, while a low muscle strength (LMS) was determined based on the lowest quintile of the HGS/BMI. Sarcopenia was defined by the coexistence of an LMM and LMS. **Results:** NAFLD was identified using a fatty liver index > 30. The participants with NAFLD had significantly higher plasma LECT2 levels compared to their non-NAFLD counterparts (34.4 [29.3–41.1] vs. 29.0 [24.7–36.7] ng/mL, *p* < 0.001). Circulating LECT2 levels were inversely correlated with ASM/BMI (*r* = −0.506, *p* < 0.001) and HGS/BMI (*r* = −0.474, *p* < 0.001), as determined by Spearman correlation analysis. Among the study participants, 79 (19.8%) were categorized as having either an LMM or LMS, and 31 (7.8%) were identified as having sarcopenia. In multivariate logistic regression, the highest LECT2 quartile had markedly greater odds of an LMM (OR 3.31, 95% CI 1.41–7.75), LMS (OR 2.85, 95% CI 1.29–6.26), and sarcopenia (OR 5.48, 95% CI 1.57–19.05) relative to the lowest quartile. **Conclusions:** Our results indicate that elevated plasma LECT2, a hepatokine increased in NAFLD, contributes to an increased risk of sarcopenia in older adults.

## 1. Introduction

Sarcopenia involves the progressive decline in muscle mass and function and is linked to various adverse health outcomes [1]. It is projected to impact approximately 10% to 16% of older adults aged > 65 years old worldwide, although its prevalence varies among studies depending on the criteria used for its definition [2]. Muscle weakness, primarily due to a diminished muscle quality rather than muscle atrophy, has led to the recognition of sarcopenia being defined more by a low muscle strength (LMS) than by a low muscle mass (LMM) [3,4]. Based on the Delphi consensus by the Global Leadership Initiative in Sarcopenia (GLIS), the muscle quantity and muscle strength were recognized as core components of the condition, while a reduced physical performance was considered as a consequence rather than a defining feature [5]. Additionally, non-alcoholic fatty liver disease (NAFLD) has become the leading contributor to chronic liver disease, impacting approximately 25% of the global population [6], with its prevalence rising rapidly owing to the increasing obesity rates and expanding elderly population [7]. NAFLD contributes to a heightened risk of metabolic disorders, including glucose intolerance and cardiovascular disease, by promoting insulin resistance, central adiposity, and lipid profile abnormalities [8].

Growing evidence suggests the link between sarcopenia and NAFLD. The potential mechanisms include aging, dietary patterns, physical inactivity, insulin resistance, chronic inflammation, vitamin D deficiency, and several hepatokines [9,10,11]. As the skeletal muscle is the key metabolic organ involved in insulin-stimulated glucose utilization, sarcopenia promotes insulin resistance regardless of obesity status [12]. Both observational and subsequent longitudinal studies have consistently demonstrated a significantly increased risk of NAFLD among patients with either an LMM or LMS, including our previous study [13,14,15,16]. Our recent nationwide, multicenter prospective study revealed NAFLD as a predictor of LMM and LMS development [17]. The role of NAFLD in the development of an LMM and LMS can be partially attributed to the secretion of various hepatokines [10,18,19].

Leucocyte cell-derived chemotaxin-2 (LECT2) was first recognized as a chemotactic factor for neutrophils; however, it has also been implicated in NAFLD, skeletal muscle insulin sensitivity, and atherosclerosis as a hepatokine [20,21]. LECT2 is upregulated in response to overnutrition, and its circulating levels are significantly higher in patients with obesity and NAFLD [20,21]. By contrast, LECT2 knock-out mice exhibited enhanced insulin responsiveness in skeletal muscle [20]. Despite the fact that LECT2 can be a link between NAFLD and sarcopenia, no human studies have explored the relationship between circulating LECT2 levels and an LMM or LMS. Therefore, this study aimed to investigate whether plasma LECT2 levels are associated with the muscle mass and strength in older Korean adults. Additionally, we aimed to examine the risk of an LMM, LMS, and sarcopenia (defined as the presence of both an LMM and LMS) based on the plasma LECT2 levels.

## 2. Materials and Methods

### 2.1. Study Population

The study population was derived from the Korean Frailty and Aging Cohort Study (KFACS), a national multicenter cohort study conducted across 10 medical centers nationwide [22]. Independently living adults aged 70–84 years were recruited at each center through age- and sex-stratified quota sampling. The baseline survey was conducted between 2016 and 2017. This study utilized data from the KFACS participants in 2017, initially including 1455 individuals. Participants were excluded based on the following criteria: use of BIA instead of DXA for body composition measurement (n = 289), excessive alcohol intake (>14 drinks per week for men and >7 for women) (n = 47), elevated liver enzyme levels (aspartate aminotransferase (AST) and alanine aminotransferase (ALT) levels > twice the upper normal limit) (n = 3), the presence of viral hepatitis (hepatitis B and hepatitis C) (n = 13), impaired renal function (creatinine level > 1.5 mg/dL) (n = 17), active inflammatory conditions (white blood cell count > 10,000/µL) (n = 14), or a history of any type of cancer (n = 36). Following these exclusions, the study included 1036 participants. Data from 2 out of 10 community health centers were excluded owing to systematic bias in the appendicular lean mass measurements obtained using bioelectrical impedance analysis compared with DXA. Among the remaining 1036 participants, 400 individuals were randomly selected for the assessment of plasma LECT2 levels (Figure S1). A post-hoc power analysis evaluating the correlations between LECT2 and muscle mass or muscle strength indices demonstrated that the statistical power to detect these correlations exceeded 0.8.

### 2.2. Measurement of the Skeletal Muscle Mass and Muscle Strength

The body composition was assessed using the Lunar (GE Healthcare, Madison, WI, USA) and Hologic (Hologic Inc., Bedford, MA, USA) DXA devices. The appendicular skeletal muscle mass (ASM), defined as the sum of the muscle masses of the four limbs, was calculated by subtracting the bone mass from the lean mass of the extremities. The muscle mass index was calculated by dividing the ASM by the body mass index (BMI) following the Foundation for the National Institutes of Health (FNIH) Sarcopenia Project’s guidelines [23]. The hand grip strength (HGS) was assessed using a digital handgrip dynamometer (Takei TKK 5401; Takei Scientific Instruments Co. Ltd., Tokyo, Japan). The grip strength in each hand was measured twice, and the greater value was considered for analysis. The muscle strength index was determined by dividing the HGS by the BMI, as this measure is a more reliable predictor of the metabolic profile than the absolute HGS value [24]. The lowest sex-specific quintiles of the ASM/BMI and HGS/BMI in the study population served as cutoff points for the LMM and LMS classification, respectively [17,25,26]. The LMM cutoff values were 0.678 for men and 0.468 for women, while the LMS cutoff values were 1.246 and 0.768 for men and women, respectively. Sarcopenia was characterized by the coexistence of both an LMM and LMS based on the revised European consensus [3,5].

### 2.3. Clinical and Biochemical Parameters

After fasting for a minimum of 8 h, blood samples were drawn at around 8 am in each center and delivered to the core laboratory (Seegene Inc., Seoul, Korea). Plasma samples were isolated via centrifugation and preserved at −80 °C before analysis. Blood chemistry tests were conducted using a Cobas 8000 C702 analyzer (Roche Diagnostics, Mannheim, Germany) except for plasma insulin and hemoglobin A1c (HbA1c) estimation. Plasma insulin was analyzed using the Cobas 8000 e602 analyzer (Roche Diagnostics, Mannheim, Germany) and HbA1c was measured using the Tosoh HLC-723 G8 analyzer (Tosoh Corporation, Tokyo, Japan). Data on smoking status, alcohol consumption, and medical conditions such as hypertension, dyslipidemia, and diabetes mellitus were collected through on-site interviews.

An NAFLD diagnosis was based on the fatty liver index (FLI), a noninvasive tool for assessing hepatic steatosis [27]. The FLI was determined using the following formula:

FLI = e^x1+e^x × 100where x=0.953×logtriglycerides+0.139×BMI                           +0.718                           ×loggamma glutamyl transferase                           +0.053×waist circumference                           −15.745

An FLI of <30 rules out hepatic steatosis detected via ultrasonography [27].

Plasma LECT2 levels were determined using a quantitative sandwich enzyme-linked immunosorbent assay kit (catalog No. RD191370200R; BioVendor, Brno, Czech Republic); the intra-assay variation coefficient was 1.6–4.3%. LECT2 levels were measured from plasma samples that had been collected in 2017 and preserved at −80 °C before analysis.

### 2.4. Statistical Analysis

Data were presented as the means ± standard deviations or median (interquartile range) for continuous variables, and as counts and percentages (%) for categorical variables. The baseline characteristics between men and women, as well as between the participants with and without an LMM, LMS, or sarcopenia, were compared using an independent two-sample *t*-test or a Mann–Whitney U-test for continuous variables and the χ^2^ test for categorical variables. Spearman correlation analysis was conducted to examine the relationship between plasma LECT2 levels and metabolic variables. Multivariate logistic regression analysis with orthogonal polynomial contrasts was performed to determine the independent association between LECT2 levels and the presence of an LMM, LMS, and sarcopenia after adjusting for age; sex; smoking status; alcohol consumption; systolic blood pressure (SBP); and total cholesterol, fasting plasma glucose (FPG), creatinine, homeostasis model assessment for insulin resistance (HOMA-IR), high sensitivity C-reactive protein (hs-CRP), and vitamin D levels with odds ratios (ORs) and the corresponding 95% confidence intervals (CIs). Data were analyzed utilizing the SPSS software (version 20.0; SPSS Inc., Chicago, IL, USA). A *p* value of <0.05 was considered significant. All statistical analyses were performed by an experienced professional.

## 3. Results

### 3.1. Baseline Characteristics

Of the 400 study participants, 189 were men, while 211 were women. Table 1 presents the baseline characteristics of the study population by sex. Men had a lower BMI but a higher waist circumference than women. Men had higher levels of gamma glutamyl transferase and creatinine and lower HOMA-IR, total cholesterol, low-density lipoprotein cholesterol, high-density lipoprotein cholesterol, and triglyceride levels than women. The prevalence of current smoking and regular alcohol consumption was higher among men than among women. The incidence rates of hypertension and dyslipidemia were lower in men, while the rates of type 2 diabetes and NAFLD were similar between the sexes. The ASM, ASM/BMI, HGS, and HGS/BMI values were greater in men compared to women. The plasma LECT2 levels were lower in men than in women (median [interquartile range]: 27 [23.7–32] vs. 36.5 [30.7–41.5] pg/mL, *p* < 0.001) (Table 1, Figure S2A). The participants with NAFLD showed higher plasma LECT2 levels compared with those without NAFLD (34.4 [29.3–41.4] pg/mL vs. 29 [24.7–36.7] pg/mL, *p* < 0.001) (Figure S2B).

### 3.2. Correlation

**Figure 1:**
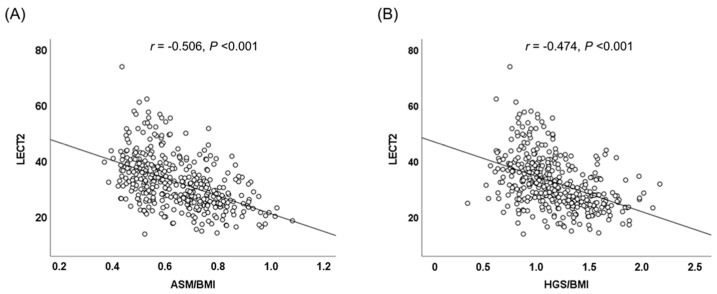
Scatter plots and Spearman correlation coefficients of the association between plasma LECT2 levels and (**A**) skeletal muscle mass index and (**B**) muscle strength index. Spearman correlation coefficients (r) and the corresponding P values are indicated. Abbreviation: ASM, appendicular skeletal muscle; BMI, body mass index; HGS, hand grip strength; LECT2, leukocyte cell-derived chemotaxin-2.

The associations between plasma LECT2 levels and clinical and metabolic parameters were examined through Spearman correlation analysis (Table 2; Figure 1). The plasma LECT2 levels were positively correlated with the BMI, waist circumference, SBP, total cholesterol, triglyceride, FPG, HOMA-IR, and hs-CRP levels and negatively correlated with the creatinine levels. Conversely, circulating LECT2 levels were inversely associated with ASM/BMI (*r* = −0.506, *p* < 0.001) and HGS/BMI (*r* = −0.474, *p* < 0.001).

**Figure 2:**
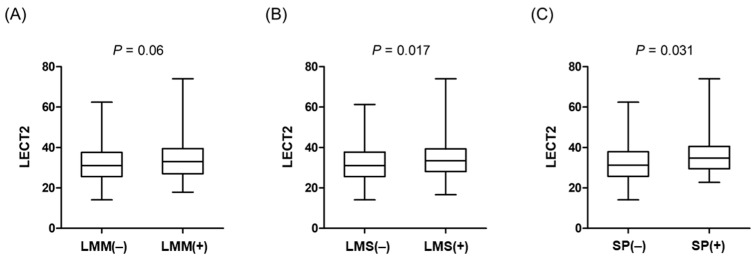
Box-and-whisker plots of plasma LECT2 levels according to (**A**) low muscle mass, (**B**) low muscle strength, and (**C**) sarcopenia. Comparisons were performed using Mann–Whitney U-test. Abbreviations: LECT2, leukocyte cell-derived chemotaxin-2; LMM, low muscle mass; LMS, low muscle strength; SP, sarcopenia.

Among the study participants, 79 (19.8%) were categorized as having either an LMM based on the ASM/BMI levels or an LMS based on the HGS/BMI levels, while 31 (7.8%) were identified as having sarcopenia, defined by the coexistence of an LMM and LMS. The baseline characteristics of the study population are described according to the presence of an LMM (Table S1), LMS (Table S2), and sarcopenia (Table S3). The participants with an LMM, LMS, and sarcopenia tended to be older and exhibited a higher BMI and waist circumference than those without these conditions. The BP level, lipid profiles, the prevalence of hypertension, type 2 diabetes, and dyslipidemia, as well as the proportion of current smokers and regular drinkers, remained comparable regardless of the LMM, LMS, or sarcopenia status. The participants with an LMM had higher plasma LECT2 levels compared with those without an LMM (33 [27–39.5] vs. 31 [25.6–37.6] pg/mL, *p* = 0.06), although this difference was not significant (Figure 2A; Table S1). In addition, the participants with LMS had significantly higher plasma LECT2 levels compared with those without an LMS (33.4 [28.1–39.3] vs. 30.8 [25.6–37.8] pg/mL, *p* = 0.017) (Figure 2B; Table S2). Additionally, the plasma LECT2 levels were elevated in the individuals with sarcopenia compared to those without the condition (34.8 [29.5–40.6] vs. 31.3 [25.8–37.9] pg/mL, *p* = 0.031) (Figure 2C; Table S3).

### 3.3. Independent Association

The independent association between LECT2 levels and the risks of LMM, LMS, and sarcopenia was evaluated using multivariate logistic regression analysis after adjusting for age; sex; smoking status; alcohol consumption; and SBP, total cholesterol, FPG, creatinine, HOMA-IR, hs-CRP, and vitamin D levels (Table 3). As the quartile of LECT2 increased, the risk of an LMM also increased. The participants in the highest quartile of LECT2 had a significantly increased risk of an LMM (OR: 3.31, 95% CI: 1.41–7.75), as did those in the third quartile (OR: 3.12, 95% CI: 1.37–7.11) and the second quartile (OR: 2.59, 95% CI: 1.13–5.95), compared with those in the lowest quartile. Additionally, the participants in the highest quartile of LECT2 had a significantly greater likelihood of an LMS (OR: 2.85, 95% CI: 1.29–6.26), as did those in the third quartile (OR: 2.32, 95% CI: 1.07–5.05), compared with the participants in the lowest quartile, while significance was not observed for those in the second quartile. Moreover, the highest quartile of LECT2 was significantly associated with the risk of sarcopenia (OR: 5.48, 95% CI: 1.57–19.05) compared with the lowest quartile, while the second or third quartile of LECT2 showed no significant association with the risk of sarcopenia. This discrepancy may be attributed to the variations in statistical power and effect sizes across the quartiles.

## 4. Discussion

The present study found that the plasma LECT2 levels negatively correlated with the muscle mass and strength. Furthermore, the plasma LECT2 levels were elevated in the participants with an LMM, LMS, or sarcopenia compared to their counterparts without these conditions. Consistently, the participants in the highest quartile of LECT2 levels showed a significantly increased risk of an LMM, LMS, and sarcopenia compared with those in the lowest quartile after adjusting for potential confounding factors. To the best of our knowledge, this study is the first study to demonstrate the relationship between the plasma LECT2 levels and muscle mass and strength. Given the upregulation of LECT2 levels in NAFLD, these results indicate that LECT2 is a key factor contributing to sarcopenia in patients with NAFLD.

The prevalence of sarcopenia differs depending on the operational diagnostic criteria and cutoff points used to define an LMM and LMS [28]. Given the correlation between the muscle mass and body size, the ASM has served as a measure of the skeletal muscle mass, adjusting for anthropometric parameters including the height, weight, and BMI [3,29]. Considering the weight changes that occur with aging, the ASM/weight could potentially underestimate the sarcopenia risk in older adults [29]. The most frequently used adjustment method is the ASM/height^2^ [3,4]. In patients with a high BMI, the ASM/height^2^ index might not accurately reflect sarcopenia owing to the large amount of fat. Our previous study reported that sarcopenia, when defined using the ASM/BMI, was associated with the insulin resistance, visceral obesity, and metabolic syndrome, while sarcopenia based on the ASM/height^2^ showed no such associations [30]. These findings suggest that the ASM/BMI-defined sarcopenia shows a stronger association with cardiometabolic risk factors compared with sarcopenia defined by other muscle mass indices. A recent study also proposed that the relative HGS, represented by the HGS/BMI, serves as a better indicator of the metabolic profile than the absolute HGS measurement [24]. Therefore, in the present study, we used the ASM/BMI and HGS/BMI as indicators of the muscle mass and strength, respectively, for comparison. As ethnic- and sex-specific cutoff points, the lowest quintile for each parameter was found to be a stronger predictor of the mortality risk compared to the FNIH-recommended values [25]. We opted to utilize the sex-specific lowest quintiles of the ASM/BMI or HGS/BMI as thresholds for identifying an LMM and LMS within the study population.

LECT2 was first recognized as a chemotactic factor for neutrophils [31] and later reidentified as a hepatokine linked to obesity [20]. The human LECT2 gene is located at 5q31.1-32, and LECT2 is predominantly produced in the hepatocytes. However, it is also expressed in various tissues, such as adipocytes; cerebral nerve cells; and vascular, endothelial, and smooth muscle cells [32,33]. Several receptors such as CD209a, tyrosine kinase with immunoglobulin-like and epidermal growth factor-like domain-1, and tyrosine-protein kinase Met mediate the effects of LECT2 as a secretory factor that modulates inflammation and fibrogenesis [34]. In mice, fasting leads to a reduction in the hepatic LECT2 gene expression and circulating LECT2 protein levels. Conversely, a high-fat diet increases the LECT2 gene expression in the liver and LECT2 protein levels in the blood [7]. Specifically, the circulating LECT2 levels correlate more closely with the hepatic triglyceride content than with the total body adiposity and serve as an indicator of weight fluctuation in murine models [35]. Moreover, circulating LECT2 levels are positively correlated with the progression of obesity and NAFLD in humans [7,21]. Additionally, Lect2 deletion attenuates the muscle insulin resistance in diet-induced obese mice through the upregulation of the genes involved in myogenesis and mitochondria, while the exposure to recombinant LECT2 disrupts insulin signaling via the phosphorylation of Jun NH2-terminal kinase in C2C12 myotubes [20]. Collectively, these findings suggest that LECT2 may serve as a potential mediator linking NAFLD and sarcopenia.

The activation of ER stress and UPR pathways promotes hepatic LECT2 expression in steatotic liver, primarily mediated by ATF4-dependent transcriptional regulation [36]. Among the proteins involved in the UPR pathway, the activating transcription factor 4 (ATF4) is the sole mediator of LECT2 transcriptional upregulation in response to ER stress [36]. ATF4 is a transcriptional activator of the UPR target genes. ATF4 interacts with three predicted binding sites within the LECT2 promoter, and this interaction is enhanced under ER stress conditions. Growing evidence suggests that the UPR pathway activated by ER stress is essential for regulating the skeletal muscle mass and metabolic functions. Adaptive UPR ameliorates ER stress by improving the protein folding and restoring the calcium homeostasis within the ER. Conversely, chronic UPR activation precipitates skeletal muscle atrophy by inhibiting protein biosynthesis, activating proteolytic cascades, and inducing inflammatory responses and insulin resistance [37]. Therefore, the upregulation of ATF4 induced by ER stress in individuals with NAFLD could play a role in the progressive decline in muscle mass and strength with aging through the increased expression of LECT2.

Several human studies have explored the association between circulating LECT2 levels and metabolic disorders, providing valuable insights into its potential role in obesity, NAFLD, insulin resistance, and cardiovascular diseases. A Japanese study utilizing health check-up data reported a positive association between circulating LECT2 levels with both adiposity and the severity of insulin resistance in humans [20]. Similarly, a Korean study demonstrated that the plasma LECT2 levels are higher in individuals with both NAFLD and metabolic syndrome, with strong associations observed with abdominal obesity, serum AST/ALT level, lipid profiles, and hsCRP [21]. Consistently, our findings indicated that the plasma LECT2 levels were significantly higher in individuals with NAFLD and positively correlated with unfavorable metabolic profiles. Further supporting these observations, Zhang et al. [38] reported that patients with newly diagnosed type 2 diabetes had significantly elevated LECT2 levels, with even higher levels in those with both diabetes and obesity. Additionally, a recent study has linked LECT2 to cardiovascular disease, showing that patients with coronary artery disease exhibit increased LECT2 levels [39]. Notably, in patients experiencing acute myocardial infarction, those with elevated LECT2 levels faced a higher likelihood of major adverse cardiovascular events over a 12-month period compared to those with lower levels. Despite these associations, no prior research in humans has specifically investigated the link between circulating LECT2 levels and sarcopenia. Our study provides new perspectives on its potential role as a mediator linking NAFLD and sarcopenia.

In this study, we found that plasma LECT2 levels negatively correlated with the muscle mass and muscle strength indices. The participants with an LMM, LMS, and sarcopenia had higher plasma LECT2 levels than those without these conditions. In the multivariate logistic regression analysis, the participants with the highest quartile of LECT2 levels showed a markedly higher likelihood of an LMM, LMS, and sarcopenia compared with those in the lowest quartile. These associations persisted even after controlling for the causative contributors to both sarcopenia and NAFLD, including insulin resistance, inflammatory markers, and vitamin D levels. This study is, to our knowledge, the first to demonstrate a relationship between the plasma LECT2 levels and muscle mass and strength. Our study also revealed that the plasma LECT2 concentrations were higher in women, despite the similar prevalence of NAFLD across the sexes. This discrepancy may reflect the fact that women have a lower muscle mass and strength than men. Additionally, the close association between visceral adiposity and circulating LECT2 levels may account for the higher LECT2 levels observed in older women compared with older men [40,41].

Our study has several limitations. First, this study lacked the access to liver imaging and histological data. Therefore, we defined NAFLD using the well-validated prediction model [27,42], which is suitable for large epidemiological studies. While the terminology for NAFLD has recently been revised to MASLD, we were unable to define MASLD due to the absence of the histological or radiological confirmation of hepatic steatosis. Owing to the intrinsic nature of a cross-sectional study, determining a causal relationship between LECT2 levels and sarcopenia was not feasible. Future research should incorporate mediation analyses to elucidate the potential mechanistic pathway linking NAFLD, LECT2, and sarcopenia. Furthermore, given that the role of LECT2 may be influenced by the extent of liver fibrosis [34], additional stratified analyses based on the fibrosis severity are warranted to gain deeper insights into its impact. Third, although the upregulation of ATF4 triggered by ER stress in patients with NAFLD may contribute to the age-related progressive loss in the muscle mass and strength through the upregulation of LECT2, additional research is needed to elucidate the role of ATF4 and the specific mechanisms by which LECT2 influences sarcopenia. In addition, as the study population comprised only Korean men and women, caution should be exercised when generalizing these findings to other ethnic groups.

Nevertheless, this study has several strengths. The KFACS is a multicenter investigation conducted across 10 centers throughout Korea to enhance the representativeness of the general population. It employs community-based sampling based on predefined inclusion and exclusion criteria, and utilizes standardized procedures for high-quality clinical and laboratory data collection. In addition, the availability of extensive data on numerous potential confounding factors further supports the validity of our study results.

## 5. Conclusions

In conclusion, high plasma LECT2 levels are independently associated with an LMM, LMS, and sarcopenia in older Korean adults. Those in the highest quartile of LECT2 showed an approximately threefold increased risk of having an LMM and LMS and a fivefold increased risk of developing sarcopenia compared with those in the lowest quartile, after adjusting for the possible covariates in this study. Further research is needed to elucidate the physiological role of circulating LECT2 in individuals with sarcopenia and explore the generalizability of our findings in different ethnicities.

## Figures and Tables

**Table 1:** Baseline characteristics of study population.

	Total	Men	Women	*p*-Value
N	400	189	211	
Age (years)	75 (73, 79)	75 (73, 79)	75 (72, 79)	0.998
BMI (kg/m^2^)	24.2 (22.3, 25.7)	23.7 (22.1, 25.6)	24.4 (22.8, 25.8)	0.031
WC (cm)	87 ± 8.1	87.9 ± 7.9	86.2 ± 8.2	0.030
SBP (mmHg)	129 (119, 138.8)	129 (118.3, 138)	129.3 (119.3, 139.7)	0.483
DBP (mmHg)	76 (70.7, 81.3)	77 (71, 81.3)	75.3 (70.3, 82)	0.283
AST (IU/L)	21 (18, 25)	21 (19, 24)	21 (18, 25)	0.392
ALT (IU/L)	17 (13, 21)	16 (13, 21)	17 (13, 21)	0.946
GGT (IU/L)	18 (14, 25)	20 (17, 28)	16 (13, 22)	<0.001
FPG (mg/dL)	96 (88, 110)	96 (88, 110)	96 (88, 109)	0.908
HOMA-IR	1.4 (0.9, 2.3)	1.3 (0.8, 1.8)	1.6 (1.1, 2.8)	<0.001
HbA1c	5.8 (5.5, 6.3)	5.7 (5.4, 6.1)	5.8 (5.5, 6.4)	0.028
TC (mg/dL)	171.7 ± 35	164.4 ± 35.5	178.2 ± 33.2	<0.001
LDL-C (mg/dL)	103.5 (79.5, 125)	100 (77, 121)	108 (81, 130)	0.009
HDL-C (mg/dL)	51 (42, 60)	49 (40, 59)	53 (44, 62)	0.007
TG (mg/dL)	105 (78.5, 139.5)	96 (74, 132)	113 (83, 159)	0.001
hs-CRP (mg/dL)	0.7 (0.5, 1.2)	0.7 (0.4, 1.3)	0.7 (0.5, 1.2)	0.953
BUN (mg/dL)	16 (13, 19)	16 (14, 19)	15 (13, 18)	0.068
Creatinine (mg/dL)	0.8 (0.7, 0.9)	0.9 (0.8, 1.1)	0.7 (0.6, 0.8)	<0.001
Vitamin D (ng/mL)	22.8 (17.2, 30.8)	23.3 (18.4, 30.1)	21.9 (15.7, 31.8)	0.244
Current smoker (n, %)	26 (6.5)	24 (12.7)	2 (1)	<0.001
Regular drinking(n, %)	52 (13)	45 (23.8)	7 (3.3)	<0.001
Hypertension (n, %)	253 (63.3)	110 (58.2)	143 (67.8)	0.047
Dyslipidemia (n, %)	155 (38.8)	56 (29.6)	99 (46.9)	<0.001
Diabetes mellitus (n, %)	92 (23)	44 (23.3)	48 (22.8)	0.900
NAFLD (n, %)	142 (35.5)	68 (36)	74 (35.1)	0.850
LECT2 (ng/mL)	31.7 (25.9, 38.2)	27 (23.7, 32)	36.5 (30.7, 41.5)	<0.001
ASM (kg)	15 (12.3, 17.9)	18 (16.7, 19.7)	12.6 (11.3, 13.9)	<0.001
ASM/BMI (m^2^)	0.61 (0.52, 0.75)	0.75 (0.7, 0.82)	0.52 (0.48, 0.58)	<0.001
HGS (kg)	25.8 (21.5, 32.6)	33.1 (28.8, 36.9)	21.8 (19, 24)	<0.001
HGS/BMI (m^2^)	1.08 (0.89, 1.35)	1.36 (1.19, 1.54)	0.9 (0.78, 1.01)	<0.001

Data are reported as mean ± SD or median (interquartile range) or n (%). Comparisons were performed using independent two-sample *t*-test or Mann–Whitney U-test for continuous variables and the χ^2^ test for categorical variables. Abbreviations: ASM, appendicular skeletal muscle; ALT, alanine aminotransferase; AST, aspartate aminotransferase; BMI, body mass index; BUN, blood urea nitrogen; DBP, diastolic blood pressure; FPG, fasting plasma glucose; GGT, gamma-glutamyltransferase; HDL-C, high-density lipoprotein cholesterol; HGS, handgrip strength; HOMA-IR, homeostatic model assessment for insulin resistance index; hs-CRP, high-sensitivity C-reactive protein; LDL-C, low-density lipoprotein cholesterol; LECT2, leucocyte cell-derived chemotaxin-2; NAFLD, non-alcoholic fatty liver disease; SBP, systolic blood pressure; TC, total cholesterol; TG, triglyceride; WC, waist circumference.

**Table 2:** Spearman correlation analysis of plasma LECT2 with clinical and metabolic parameters.

	*r*	*p*
Age	−0.080	0.109
Sex (men)	−0.515	<0.001
BMI	0.261	<0.001
WC (cm)	0.156	0.002
SBP (mmHg)	0.129	0.010
GGT (IU/L)	0.068	0.172
TC (mg/dL)	0.203	<0.001
TG (mg/dL)	0.314	<0.001
FPG (mg/dL)	0.155	0.002
HOMA-IR	0.288	<0.001
Creatinine (mg/dL)	−0.245	<0.001
hs-CRP (mg/dL)	0.135	0.007
Vitamin D (ng/mL)	−0.089	0.075
ASM (kg)	−0.384	<0.001
ASM/BMI (m^2^)	−0.506	<0.001
HGS (kg)	−0.379	<0.001
HGS/BMI (m^2^)	−0.474	<0.001

Abbreviations: ASM, appendicular skeletal muscle; BMI, body mass index; FPG, fasting plasma glucose; GGT, gamma-glutamyltransferase; HGS, hand grip strength; HOMA-IR, homeostasis model assessment for insulin resistance; hs-CRP, high-sensitivity C-reactive protein; LECT2, leucocyte cell-derived chemotaxin-2; SBP, systolic blood pressure; TC, total cholesterol; TG, triglyceride.

**Table 3:** ORs and 95% CIs of low muscle mass (LMM), low muscle strength (LMS), and sarcopenia by quartiles of plasma LECT2 concentrations.

LMM	ORs (95% CIs)				
	Q1	Q2	Q3	Q4	*p* for Trend
Model 1	1 (ref)	2.53 (1.12–5.71)	3.0 (1.34–6.74)	2.96 (1.31–6.68)	0.008
Model 2	1 (ref)	2.56 (1.13–5.8)	2.97 (1.32–6.69)	3.07 (1.35–6.97)	0.007
Model 3	1 (ref)	2.54 (1.11–5.79)	3.04 (1.34–6.89)	3.22 (1.39–7.50)	0.006
Model 4	1 (ref)	2.59 (1.13–5.95)	3.12 (1.37–7.11)	3.31 (1.41–7.75)	0.005
LMS	ORs (95% CIs)				
	Q1	Q2	Q3	Q4	*p* for trend
Model 1	1 (ref)	0.83 (0.36–1.92)	2.28 (1.08–4.82)	2.86 (1.36–6.01)	0.001
Model 2	1 (ref)	0.83 (0.36–1.94)	2.28 (1.08–4.82)	2.94 (1.4–6.19)	0.001
Model 3	1 (ref)	0.83 (0.35–1.94)	2.2 (1.03–4.7)	2.73 (1.27–5.88)	0.001
Model 4	1 (ref)	0.87 (0.36–2.07)	2.32 (1.07–5.05)	2.85 (1.29–6.26)	0.001
Sarcopenia	ORs (95% CIs)				
	Q1	Q2	Q3	Q4	*p* for trend
Model 1	1 (ref)	1.55 (0.42–5.73)	1.97 (0.55–7.05)	4.78 (1.47–15.56)	0.010
Model 2	1 (ref)	1.59 (0.43–5.93)	2.1 (0.58–7.57)	5.38 (1.63–17.75)	0.006
Model 3	1 (ref)	1.59 (0.42–6.0)	2.05 (0.56–7.49)	5.0 (1.47–17.02)	0.010
Model 4	1 (ref)	1.85 (0.48–7.2)	2.32 (0.62–8.69)	5.48 (1.57–19.05)	0.008

Model 1: adjusted for age and sex. Model 2: Model 1 + smoking and alcohol. Model 3: Model 2 + SBP, TC, FPG, creatinine. Model 4: Model 3 + HOMA-IR, hs-CRP, vitamin D. Abbreviations: LECT2, leukocyte cell-derived chemotaxin-2.

## Data Availability

Qualified researchers can obtain the data from the corresponding author (medica7@korea.ac.kr). The data are not publicly available due to privacy concerns imposed by the IRB.

## Supplementary Materials

The following supporting information can be downloaded at: https://www.mdpi.com/article/10.3390/nu17081342/s1, Table S1. Baseline characteristics of study population according to low muscle mass (LMM); Table S2. Baseline characteristics of study population according to low muscle strength (LMS); Table S3. Baseline characteristics of study population according to sarcopenia; Figure S1. Flowchart of participant selection for the analysis; Figure S2. Box-and-whisker plots of plasma LECT2 levels according to (A) sex, (B) nonalcoholic fatty liver disease defined by the fatty liver index score.
