# Supplementary material for: Plasma Leukocyte Cell-Derived Chemotaxin-2 as a Risk Factor of Sarcopenia: Korean Frailty and Aging Cohort Study
Source: Nutrients. 2025 Apr 14;17(8):1342. doi: 10.3390/nu17081342 (PMC12029840; doi:10.3390/nu17081342)
Supplement: Supplementary file 1 [file nutrients-17-01342-s001.zip › nutrients-3484245-supplementary.pdf]

Plasma Leukocyte Cell-Derived Chemotaxin-2 as a Risk Factor of Sarcopenia: Korean Frailty and Aging Cohort Study

Supplementary Materials

Contents:

Table S1. Baseline characteristics of study population according to low muscle mass (LMM)

Table S2. Baseline characteristics of study population according to low muscle strength (LMS)

Table S3. Baseline characteristics of study population according to sarcopenia

Figure S1. Flowchart of participant selection for the analysis

Figure S2. Box-and-whisker plots of plasma LECT2 levels according to (A) sex, (B) nonalcoholic fatty liver disease defined by the fatty liver index score

Table S1. Baseline characteristics of study population according to low muscle mass (LMM)

	No LMM	LMM	
N	321	79	*P*-value
Age (years)	75 (72,79)	76 (74,80)	0.015
Men	152 (47.4)	37 (46.8)	0.934
BMI (kg/m2)	24 ± 2.7	25.1 ± 2.4	0.001
WC (cm)	86.5 ± 8	89.3 ± 8	0.005
SBP (mmHg)	129 (119,139.3)	127.7 (117.3,136.7)	0.390
DBP (mmHg)	76.3 (71.3,81.3)	74 (68,81.3)	0.096
AST (IU/L)	21 (19,25)	21 (17,24)	0.496
ALT (IU/L)	17 (13,21)	17 (13,20)	0.412
GGT (IU/L)	18 (14,26)	18 (14,24)	0.836
FPG (mg/dL)	97 (88,110)	94 (88,106)	0.568
HOMA-IR	1.4 (0.9,2.3)	1.5 (0.9,2.1)	0.988
HbA1c	5.8 (5.5,6.2)	5.7 (5.4,6.3)	0.735
TC (mg/dL)	171 ± 35.5	174.4 ± 32.8	0.442
LDL-C (mg/dL)	103 (79,125)	107 (81,125)	0.638
HDL-C (mg/dL)	51 (42,60)	53 (44,64)	0.191
TG (mg/dL)	104 (78,138)	107 (82,145)	0.503
hs-CRP (mg/dL)	0.7 (0.4,1.3)	0.7 (0.5,1.1)	0.899
BUN (mg/dL)	15 (13,19)	17 (15,19)	0.009
Creatinine (mg/dL)	0.8 (0.7,0.9)	0.8 (0.7,1)	0.703
Vitamin D (ng/ml)	22.9 (17.5,30.9)	22.1 (16.5,29.6)	0.451
LECT2 (ng/ml)	31 (25.6,37.6)	33 (27,39.5)	0.060
Current smoker (n, %)	20 (6.2)	6 (7.6)	0.659
Regular drinking(n, %)	45 (14)	7 (8.9)	0.222
Hypertension (n, %)	200 (62.3)	53 (67.1)	0.430
Dyslipidemia (n, %)	121 (37.7)	34 (43)	0.383
Diabetes mellitus (n, %)	74 (23.1)	18 (22.8)	0.960
NAFLD (n, %)	109(34)	33(41.8)	0.193
ASM (kg)	15.4 (12.9,18.3)	12.2 (10.8,16.4)	<0.001
ASM/BMI (m2)	0.63 (0.54,0.76)	0.46 (0.44,0.66)	<0.001
HGS (kg)	26.3 (21.8,32.8)	23.7 (20.1,30.7)	0.032
HGS/BMI (m2)	1.09 (0.92,1.39)	0.96 (0.76,1.23)	0.001

Data are presented as mean ± SD or median (interquartile range) or n (%). Comparisons were performed using independent two-sample t-test or Mann-Whiney U-test for continuous variables and the χ^2 test for categorical variables. Abbreviation: ASM, appendicular skeletal muscle; ALT, alanine aminotransferase; AST, aspartate aminotransferase; BMI, body mass index; BUN, blood urea nitrogen; DBP, diastolic blood pressure; FPG, fasting plasma glucose; GGT, gamma-glutamyltransferase; HDL-C, high-density lipoprotein cholesterol; HGS, handgrip strength; HOMA-IR, homeostatic Model Assessment for Insulin resistance index; hs-CRP, high sensitivity C-reactive protein; LDL-C, low-density lipoprotein cholesterol; LECT2, leucocyte cell-derived chemotaxin-2; NAFLD, non-alcoholic fatty liver disease; SBP, systolic blood pressure; TC, total cholesterol; TG, triglyceride; WC, waist circumference.

Table S2. Baseline characteristics of study population according to low muscle strength (LMS)

	No LMS	LMS	
N	321	79	*P*-value
Age (years)	75 (72,78)	77 (74,81)	<0.001
Men	152 (47.4)	37 (46.8)	0.934
BMI (kg/m2)	23.9 ± 2.4	25.5 ± 3	<0.001
WC (cm)	85.9 ± 7.6	91.7 ± 8.2	<0.001
SBP (mmHg)	128.3 (117.7,138)	132.7 (122,141)	0.053
DBP (mmHg)	76.2 ± 8.8	76.9 ± 9.1	0.548
AST (IU/L)	21 (18,24)	21 (19,25)	0.282
ALT (IU/L)	16 (13,21)	17 (13,21)	0.539
GGT (IU/L)	18 (14,26)	19 (15,24)	0.337
FPG (mg/dL)	95 (88,109)	99 (91,111)	0.098
HOMA-IR	1.4 (0.9,2.2)	1.6 (1.1,2.7)	0.033
HbA1c	5.7 (5.5,6.2)	5.8 (5.5,6.3)	0.515
TC (mg/dL)	172.2 ± 35.3	169.7 ± 33.8	0.567
LDL-C (mg/dL)	104 (79,128)	102 (82,123)	0.923
HDL-C (mg/dL)	52 (43,60)	50 (41,59)	0.488
TG (mg/dL)	104 (78,137)	109 (82,148)	0.249
hs-CRP (mg/dL)	0.7 (0.4,1.2)	0.8 (0.5,1.5)	0.042
BUN (mg/dL)	16 (13,19)	17 (14,18)	0.422
Creatinine (mg/dL)	0.8 (0.7,0.9)	0.8 (0.7,1)	0.436
Vitamin D (ng/ml)	22.9 (17.5,30.8)	22.6 (17.2,30.8)	0.758
LECT2 (ng/ml)	30.8 (25.6,37.8)	33.4 (28.1,39.3)	0.017
Current smoker (n, %)	21 (6.5)	5 (6.3)	0.945
Regular drinking(n, %)	44 (13.7)	8 (10.1)	0.397
Hypertension (n, %)	197 (61.4)	56 (70.9)	0.116
Dyslipidemia (n, %)	121 (37.7)	34 (43)	0.383
Diabetes mellitus (n, %)	70 (21.8)	22 (27.9)	0.253
NAFLD (n, %)	97 (30.2)	45 (57)	<0.001
ASM (kg)	14.9 (12.6,17.9)	15.3 (11.9,17.7)	0.283
ASM/BMI (m2)	0.62 (0.53,0.75)	0.56 (0.47,0.7)	0.001
HGS (kg)	27.1 (22.4,33.7)	20.4 (17,26.6)	<0.001
HGS/BMI (m2)	1.14 (0.95,1.43)	0.74 (0.65,1.07)	<0.001

Data are presented as mean ± SD or median (interquartile range) or n (%). Comparisons were performed using independent two-sample t-test or Mann-Whiney U-test for continuous variables and the χ^2 test for categorical variables. Abbreviation: ASM, appendicular skeletal muscle; ALT, alanine aminotransferase; AST, aspartate aminotransferase; BMI, body mass index; BUN, blood urea nitrogen; DBP, diastolic blood pressure; FPG, fasting plasma glucose; GGT, gamma-glutamyltransferase; HDL-C, high-density lipoprotein cholesterol; HGS, handgrip strength; HOMA-IR, homeostatic Model Assessment for Insulin resistance index; hs-CRP, high sensitivity C-reactive protein; LDL-C, low-density lipoprotein cholesterol; LECT2, leucocyte cell-derived chemotaxin-2; NAFLD, non-alcoholic fatty liver disease; SBP, systolic blood pressure; TC, total cholesterol; TG, triglyceride; WC, waist circumference.

Table S3. Baseline characteristics of study population according to sarcopenia

	No sarcopenia	Sarcopenia	
N	369	31	*P*-value
Age (years)	75 (72,79)	77 (74,81)	0.019
Men	176 (47.7)	13 (41.9)	0.537
BMI (kg/m2)	24 ± 2.6	25.9 ± 2.7	<0.001
WC (cm)	86.6 ± 7.9	92.4 ± 7.7	<0.001
SBP (mmHg)	129 (118.3,138.7)	129.3 (122,139.7)	0.560
DBP (mmHg)	75.7 (70.7,81.3)	77.3 (68.3,84.3)	0.475
AST (IU/L)	21 (18,25)	21 (20,25)	0.291
ALT (IU/L)	17 (13,21)	17 (13,22)	0.912
GGT (IU/L)	18 (14,26)	19 (15,24)	0.793
FPG (mg/dL)	96 (88,110)	97 (92,110)	0.183
HOMA-IR	1.4 (0.9,2.2)	1.7 (1.2,3)	0.170
HbA1c	5.7 (5.5,6.2)	5.8 (5.5,6.4)	0.819
TC (mg/dL)	171.8 ± 35.3	170.5 ± 30.4	0.850
LDL-C (mg/dL)	104 (79,126)	103 (82,123)	0.724
HDL-C (mg/dL)	51 (42,60)	52 (42,64)	0.432
TG (mg/dL)	105 (78,138)	107 (84,159)	0.534
hs-CRP (mg/dL)	0.7 (0.4,1.2)	0.8 (0.5,1.5)	0.442
BUN (mg/dL)	16 (13,19)	17 (15,20)	0.059
Creatinine (mg/dL)	0.8 (0.7,0.9)	0.8 (0.7,1.1)	0.596
Vitamin D (ng/ml)	22.8 (17.2,30.7)	23.2 (18.8,35.3)	0.437
LECT2 (ng/ml)	31.3 (25.8,37.9)	34.8 (29.5,40.6)	0.031
Current smoker (n, %)	26 (7.1)	1 (3.2)	0.246
Regular drinking(n, %)	51 (13.8)	1 (3.2)	0.158
Hypertension (n, %)	231 (62.6)	22 (71)	0.353
Dyslipidemia (n, %)	138 (37.4)	17 (54.8)	0.056
Diabetes mellitus (n, %)	84 (22.8)	8 (25.8)	0.699
NAFLD (n, %)	123 (33.3)	19 (61.3)	0.002
ASM (kg)	15.1 (12.7,18)	12.2 (10.8,16.4)	0.001
ASM/BMI (m2)	0.62 (0.52,0.75)	0.44 (0.42,0.65)	<0.001
HGS (kg)	26.4 (21.8,33.1)	18.8 (17.6,26.9)	<0.001
HGS/BMI (m2)	1.09 (0.91,1.37)	0.73 (0.69,1.05)	<0.001

Data are presented as mean ± SD or median (interquartile range) or n (%). Comparisons were performed using independent two-sample t-test or Mann-Whiney U-test for continuous variables and the χ^2 test for categorical variables. Abbreviation: ASM, appendicular skeletal muscle; ALT, alanine aminotransferase; AST, aspartate aminotransferase; BMI, body mass index; BUN, blood urea nitrogen; DBP, diastolic blood pressure; FPG, fasting plasma glucose; GGT, gamma-glutamyltransferase; HDL-C, high-density lipoprotein cholesterol; HGS, handgrip strength; HOMA-IR, homeostatic Model Assessment for Insulin resistance index; hs-CRP, high sensitivity C-reactive protein; LDL-C, low-density lipoprotein cholesterol; LECT2, leucocyte cell-derived chemotaxin-2; NAFLD, non-alcoholic fatty liver disease; SBP, systolic blood pressure; TC, total cholesterol; TG, triglyceride; WC, waist circumference.

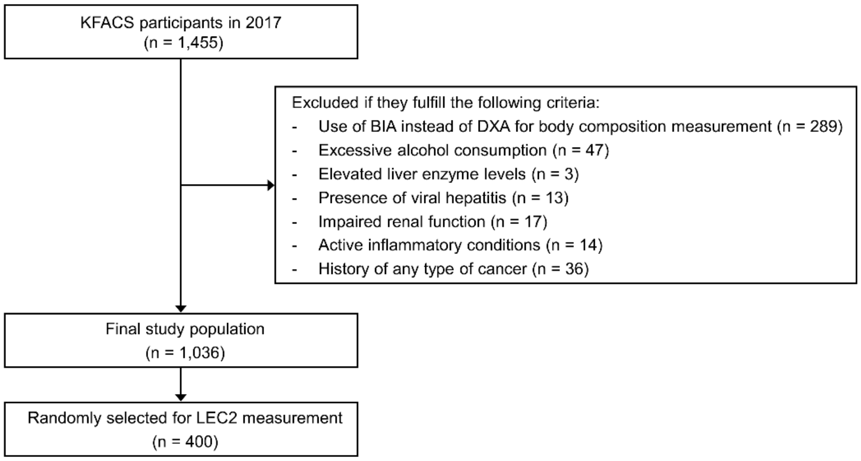

Figure S1. Flowchart of participant selection for the analysis

Abbreviation: KFACS, Korean Frailty and Aging Cohort Study; LECT2, leukocyte cell-derived chemotaxin-2; NAFLD, nonalcoholic fatty liver disease

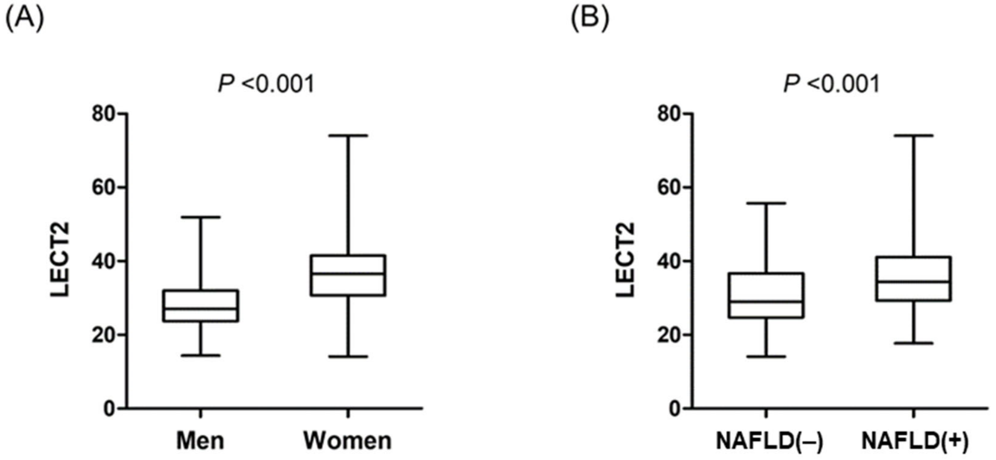

Figure S2. Box-and-whisker plots of plasma LECT2 levels according to (A) sex, (B) nonalcoholic fatty liver disease defined by the fatty liver index score

Comparisons were performed using Mann-Whitney U-test. Abbreviation: LECT2, leukocyte cell-derived chemotaxin-2; NAFLD, nonalcoholic fatty liver disease
